#  Sarcoptic mange in wild ruminants in Spain: solving the epidemiological enigma using microsatellite markers

**DOI:** 10.1186/s13071-021-04673-x

**Published:** 2021-03-20

**Authors:** Barbara Moroni, Samer Angelone, Jesús M. Pérez, Anna Rita Molinar Min, Mario Pasquetti, Paolo Tizzani, Jorge Ramón López-Olvera, Marta Valldeperes, José Enrique Granados, Santiago Lavín, Gregorio Mentaberre, Leonor Camacho-Sillero, Carlos Martínez-Carrasco, Alvaro Oleaga, Mónica Candela, Pier Giuseppe Meneguz, Luca Rossi

**Affiliations:** 1 Department of Veterinary Science, University of Turin, Largo Braccini 2, 10095 Grugliasco, Italy; 2 Department of Evolutionary Biology and Environmental Studies, University of Zurich, Zurich, Switzerland; 3 Department of Animal and Plant Biology, and Ecology, University of Jaén, Campus Las Lagunillas, Jaén, Spain; 4 Wildlife Ecology & Health Group (WE&H), and Departament de Medicina I Cirurgia Animals, Universitat Autònoma de Barcelona, Cerdanyola del Vallès, Spain; 5 Wildlife Ecology & Health Group (WE&H), and Parque Nacional Y Parque Natural Sierra Nevada, Granada, Spain; 6 Wildlife Ecology & Health Group (WE&H), and Departament de Ciència Animal, Escola Tècnica Superior D’Enginyeria Agraria, Universitat de Lleida, Lleida, Spain; 7 Programa Vigilancia Epidemiológica Fauna Silvestre, Consejería Agricultura, Ganadería, Pesca y Desarrollo Sostenible, Junta de Andalucía, Málaga, Spain; 8 Departamento de Sanidad Animal, Facultad de Veterinaria, Universidad de Murcia, Campus de Espinardo, 30100 Murcia, Spain; 9 S.E.R.P.A., Sociedad de Servicios del Principado de Asturias S.A., Gijón, Spain

**Keywords:** *Sarcoptes scabiei*, Ruminant populations, Spain, Wildlife, Molecular markers, Molecular epidemiology, Host specificity, Genetic structure

## Abstract

**Background:**

In Spain, sarcoptic mange was first described in native wildlife in 1987 in Cazorla Natural Park*,* causing the death of nearly 95% of the local native population of Iberian ibex (*Capra pyrenaica*). Since then, additional outbreaks have been identified in several populations of ibex and other wild ungulate species throughout the country. Although the first epizootic outbreak in wildlife was attributed to the introduction of an infected herd of domestic goats, the origin and the cause of its persistence remain unclear. The main aims of this study are to understand (i) the number of *Sarcoptes scabiei “*strains” circulating in wild ruminant populations in Spain, and (ii) the molecular epidemiological relationships between *S. scabiei* and its hosts.

**Methods:**

Ten *Sarcoptes* microsatellite markers were used to characterize the genetic structure of 266 mites obtained from skin scrapings of 121 mangy wild ruminants between 2011 and 2019 from 11 areas in Spain.

**Results:**

Seventy-three different alleles and 37 private alleles were detected. The results of this study show the existence of three genetic strains of *S. scabiei* in the wild ruminant populations investigated. While two genetic clusters of *S. scabiei* were host- and geography-related, one cluster included multi-host mites deriving from geographically distant populations.

**Conclusions:**

The molecular epidemiological study of *S. scabiei* in wild ruminants in Spain indicates that the spreading and persistence of the parasite may be conditioned by host species community composition and the permissiveness of each host population/community to the circulation of individual “strains,” among other factors. Wildlife–livestock interactions and the role of human-driven introduction or trade of wild and domestic animals should be better investigated to prevent further spread of sarcoptic mange in as yet unaffected natural areas of the Iberian Peninsula.

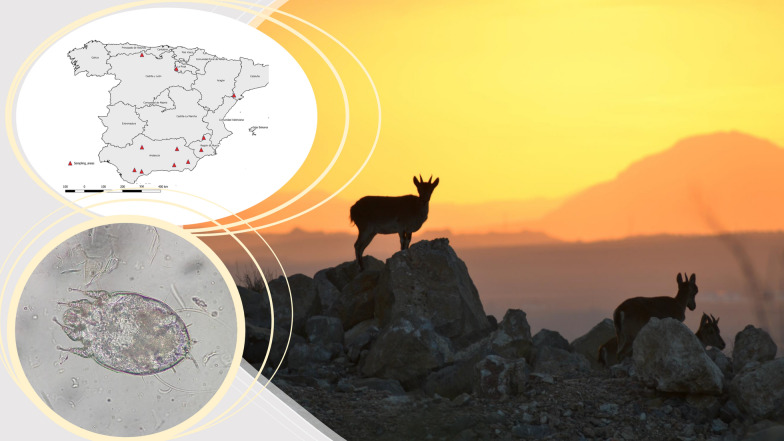

**Supplementary Information:**

The online version contains supplementary material available at 10.1186/s13071-021-04673-x.

## Background

The ubiquitous mite *Sarcoptes scabiei* affects more than 100 mammalian species worldwide, causing a highly contagious skin disease known as sarcoptic mange or scabies. Since it can result in significant declines in local wildlife populations, this disease has received particular attention in wildlife conservation and management for decades [1].

In Spain, sarcoptic mange was first described in native free-ranging wild ruminants in late 1987 in the Cazorla Natural Park [2]*,* causing a decline of nearly 95% in the local native population of Iberian ibex (*Capra pyrenaica*) over 4 years [3]. Since then, additional mange outbreaks have been identified in other wild ungulate populations, including red deer (*Cervus elaphus*) [4], Cantabrian chamois (*Rupicapra pyrenaica parva*) [5, 6], fallow deer (*Dama dama*) [7], roe deer (*Capreolus capreolus*) [8], European mouflon (*Ovis aries musimon*) [2], and the non-native Barbary sheep (*Ammotragus lervia*) [9]. Whereas high mortality rates and associated population declines have been recorded in Cantabrian chamois and Iberian ibex [2], in the remaining species the infection seems to be less deleterious [4, 10].

Since the direct life cycle of *S. scabiei* relies on suitable hosts, a multi-host system can provide the parasite with higher opportunities to persist and spread [11]. Wild ruminants in Spain share habitat with different recognized wild hosts for *S. scabiei*, such as red fox and Iberian wolf (*Vulpes vulpes* and *Canis lupus signatus*, respectively) [12] and wild boar (*Sus scrofa*). While foxes are scavengers and might represent only a marginal and weak transmission pattern of sarcoptic mange for wild cervids and bovids, top predators such as the Iberian wolf, which is currently present in Spain mostly in the northern region (Asturias, Cantabria), might have prey–predator interactions with red and roe deer and, on rare occasions, with chamois [13, 14]. Interestingly, sarcoptic mange episodes in wild boar in Spain have never been reported in the scientific literature, although serological positivity to *S. scabiei* has been detected [15], and wildlife operators have occasionally reported crusted lesions compatible with sarcoptic mange in wild boars.

Although morphological studies of *S. scabiei* mites have failed to recognize host-specific differences [16, 17], epidemiological and pathological findings have detected geographical and host-specific patterns of mange epidemics in wildlife [10, 12, 18]. In turn, these findings and growing molecular epidemiological data [19–22] have called into question the traditional, still widely accepted, classification of *S. scabiei* into species-specific variants [11]. Recently, the use of molecular markers such as microsatellites (known as short tandem repeats [STR] or simple sequence repeats [SSR]) has revealed the existence of host-specific genetic “strains.” While the traditional classification of species-specific variants is based on clinical, epidemiological, and biological criteria, host-specific strains are based on population genetic criteria that clearly identified differences between sarcoptic mange outbreaks in various animal species and areas. In particular, two main transmission models based on genetic structure were proposed, namely the “host–taxon” law [19] and the “prey-to-predator” interaction [20]. A third model revealing a possible cryptic transmission of *S. scabiei* between raccoon dogs and Japanese serow with weak prey–predator interaction (in contrast to the strong prey-to-predator interaction highlighted between cheetahs and Thompson gazelle in Kenya [20]) has also been proposed [21]. All the models rely on the assumption that close contact between different host species within the same habitat is possible and may result in effective multi-host transmission of *S. scabiei*. Environmental transmission is also a viable transmission model for sarcoptic mange in wild ungulates, as hypothesized by several authors in typical resting sites such as caves in Cazorla Natural Park [2] and in salt lick sites in the Alps [23], where the frequent and alternative transition of infected animals, or even the presence of infected carcasses, might favor the indirect transmission of the disease. Although the first epizootic outbreak reported in wildlife in the Iberian Peninsula was attributed to the introduction of an infected herd of domestic goats [2], the origin and the cause of the persistence of *S. scabiei* in wild ruminant populations are still unclear. Some decades ago, wild ungulate populations in Spain were largely evenly distributed in mountain territories, with rare interactions among free-ranging communities located in the different mountain systems. In recent years, wild ungulates have increased in both number and range in Spain, as in the rest of Europe, favored by rural abandonment, reforestation, reintroduction, and legislative changes [24]. This has connected formerly isolated populations through corridors. While the re-creation of connection has a major positive effect on biodiversity, it also can favor the spread and transmission of pathogens such as *S. scabiei*. Whether livestock or human-driven wildlife movement and introduction play a key role in the spread of this parasitosis is still an ongoing and open debate [25].

Using *S. scabiei* mites isolated from 11 populations of six wild ruminant species in Spain, this study aims to describe the genetic structure of the circulating *S. scabiei* “strains,” namely (i) the number of *S. scabiei* strains that can be molecularly identified in wild ruminant populations in Spain, and (ii) the epidemiological relationships between *S. scabiei* and the wild ruminant communities within the main outbreak areas countrywide.

## Methods

### Collection of mites

**Fig. 1:**
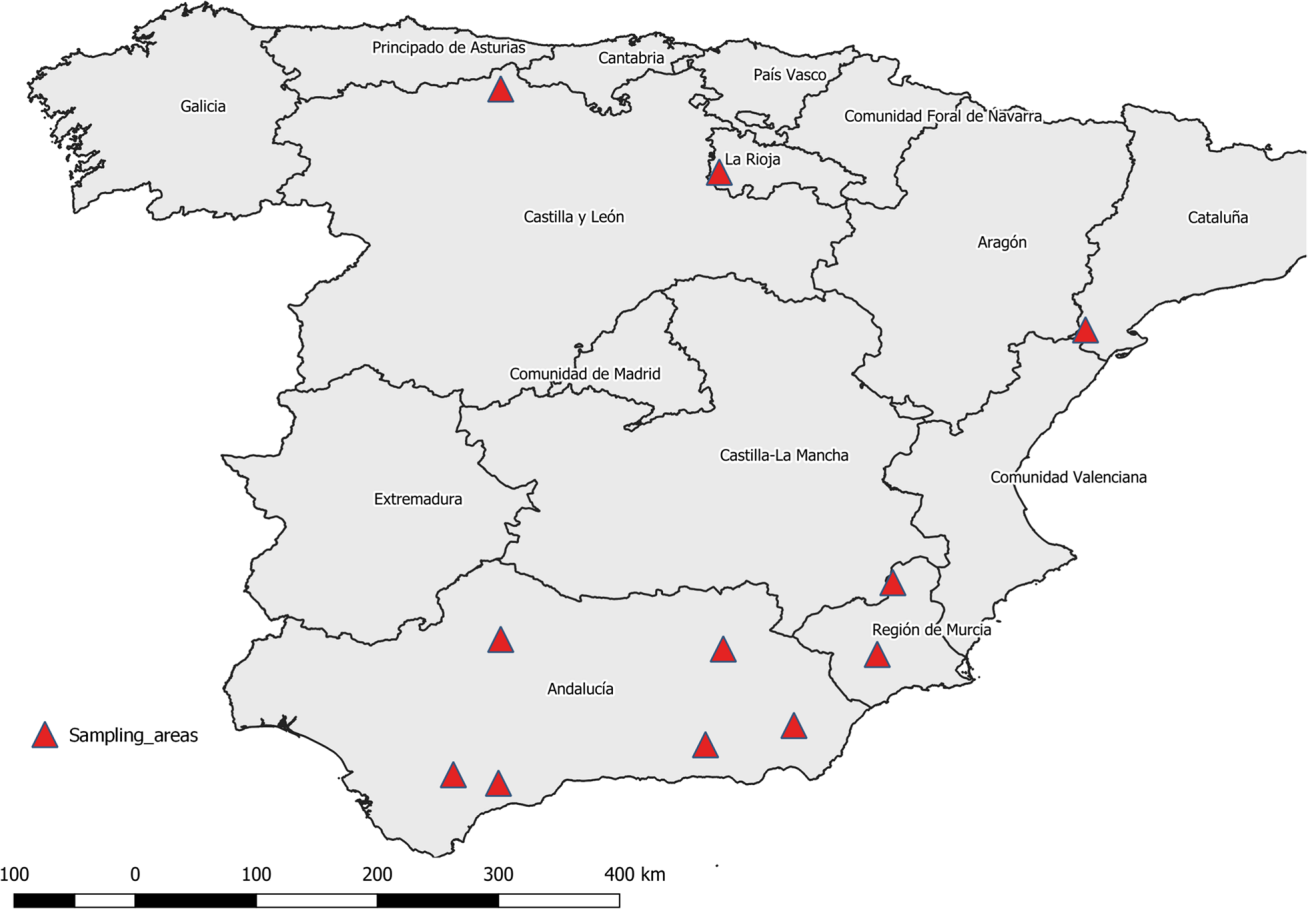
Map showing the 11 sampling sites of ruminants affected by sarcoptic mange in the Iberian Peninsula

Skin samples from 121 mangy wild ruminants were collected during regular management plan activities or seasonal culling programs between 2011 and 2019 in 11 areas in Spain (Fig. 1 and Table 1). The samples belonged to six ungulate species, namely Iberian ibex (83), Cantabrian chamois (16), red deer (18), roe deer (2), aoudad (1), and European mouflon (1) (Table 1). Skin samples were stored at −20 °C or in 70% ethanol tubes until mite isolation. For each skin sample, three mites were isolated and individually stored following the post-frozen isolation method [26]. All the mites were identified as *S. scabiei* following morphological criteria [27].

**Table 1:** Geographical origin, host species, sample size, and sampling year of the *Sarcoptes* included in this study

Geographical origin	Host species	Sampled animals	*Sarcoptes* isolated^a^	Sampling year	Index case
Sierra de Grazalema (Andalucía)	*C. pyrenaica*	4	11	2017	2011
Sierra de las Nieves (Andalucía)	*C. pyrenaica*	6	13	2017	1989
Sierra Nevada (Andalucía)	*C. pyrenaica*	47	100	2017	1992
Tortosa-Beceite (Cataluña)	*C. pyrenaica*	10	11	2018	2014
Sierra de Cazorla (Andalucía)	*C. pyrenaica*	9	18	2017	1987
	*O. aries musimon*	1	5		
	*C. elaphus*	2	10		
Sierra de los Filabres (Andalucía)	*C. pyrenaica*	3	4	2018	2012
Sierras del Noroeste (Murcia)	*C. pyrenaica*	4	8	2019	1990
Sierra Espuña (Murcia)	*A. lervia*	1	2	2019	1991
Cordillera Cantábrica (Asturias)	*R. pyrenaica*	16	40	2010	1993
	*C. capreolus*	2	4		
	*C. elaphus*	9	19		
Sierra Morena (Andalucía)	*C. elaphus*	2	4	2011	NA
Sierra de Demanda (La Rioja)	*C. elaphus*	5	17	2011	2010

Index case refers to the year of the first mange report in the wild host population of that area

*NA* not available

^a^Mites that fulfilled the required criteria for the population genetic analysis after molecular analysis

### DNA amplifications and microsatellite analysis

DNA was extracted from individual mites following the HotSHOT Plus ThermalSHOCK technique [28]. A 10× multiplex polymerase chain reaction (PCR) was then performed using ten validated primers extracted from the previously published panel [29] to target *S. scabiei* mites (Sarms 33, 34, 35, 36, 37, 38, 40, 41, 44, 45) [28, 30]. Primers were 5′ labeled with 6-FAM, VIC, NED, or PET fluorescent dye tag (Applied Biosystems, Foster City, CA, USA). Twelve microliters of PCR mixture, consisting of all primer pairs, ranging from 0.04 to 0.01 μM, 10× PCR buffer (200 mM KCl and 100 mM Tris–HCl, pH 8.0), 200 μM of each dinucleotide and 0.5 U HotStarTaq polymerase (QIAGEN, Milano, Italy), were admixed with 3 µl of individual mite DNA and subjected to thermal reactions in an Applied Biosystems 2720 thermal cycler (Applied Biosystems, Foster City, CA, USA), according to the following protocol: 15 min at 95 °C (initial denaturing), followed by 37 cycles of three steps of 30 s at 94 °C (denaturation), 45 s at 55 °C (annealing), and 1.5 min at 72 °C (extension), before a final elongation of 7 min at 72 °C. The PCR products (1 µl) were then mixed with 12 µl of formamide with GeneScan 500 LIZ Size Standard (Applied Biosystems, Foster City, CA, USA) in a 96-well plate and heated at 95 °C for 5 min. Capillary electrophoresis was performed with an ABI PRISM 310 Genetic Analyzer, and GeneMapper 4.0 software (Applied Biosystems, Foster City, CA, USA) was used for the allele calls and microsatellite visualization. After molecular analysis, only the mites that fulfilled the required criteria (eight detectable loci out of the ten analyzed) were included in the molecular analyses.

### Genetic analysis

Three main population genetics analyses were applied to the 266 mite microsatellite outputs: (i) Bayesian clustering, (ii) genetic distance (to calculate the proportion of shared alleles), and iii) principal component analysis (PCA). The first one requires Hardy–Weinberg equilibrium (HWE), while no assumptions are required for the second and third analyses.

Descriptive statistics, including observed and expected heterozygosis (Ho and He, respectively), allelic richness (R), and HWE analysis, were carried out in the R 4.0 software environment using the packages Adegenet 2.1.3 and Pegas 0.3 [31, 32].

*P* values for the HWE test were based on Monte Carlo permutations of alleles. The Bayesian assignment test was computed with STRUCTURE 2.3.4 software [33]. Burn-in and run lengths of Markov chains were 10,000 and 100,000, respectively, and ten independent runs for each *K* (for *K* = 1–20) were run. The ancestry model was selected as the admixture model. The estimation of clusters was performed as previously described [34], using the deltaK method. Individual mites were then assigned to the corresponding inferred cluster.

Genetic distances and multilocus proportion of shared alleles (DPS) among mite populations were computed between all possible pairs of individuals using microsatellite analyzer (MSA 4.0) and Populations 1.2.32 software, and then displayed with interactive Tree of Life (iTOL) [35] as unrooted dendrogram.

Multivariate analysis (PCA) was performed with R 4.0 without any preliminary assumptions on the origin of the mite samples. The populations of mites in this analysis were labeled as reported in Table 1.

## Results

Seventy-three different alleles were detected in the 266 mites isolated from the 11 wild ruminant populations using ten microsatellite loci as molecular markers (Additional file 1: Table S1). Depending on the loci, allele count ranged from three (Sarms 37) to 13 (Sarms 45). Thirty-seven private alleles (alleles found only in one population) were detected, ranging from 1 (Murcia) to 18 (La Rioja), whereas no private alleles were found in the Iberian ibexes from Tortosa Beceite, Sierra de Grazalema, Sierra de los Filabres, or Sierra de las Nieves, or in the red deer from Sierra Morena. Ho and He ranged from 0.03 (Ho) and 0.04 (He) to 0.13 (Ho) and 0.72 (He) in Sarms 44, Sarms 37, and Sarms 34, respectively (Table 2).

**Table 2:** Expected (He) and observed (Ho) heterozygosis and allelic richness (R) for each locus corresponding to the microsatellite (Sarms) number

	Sarms33	Sarms34	Sarms35	Sarms36	Sarms37	Sarms38	Sarms40	Sarms41	Sarms44	Sarms45
He	0.72	0.63	0.64	0.57	0.58	0.66	0.73	0.28	0.71	0.71
Ho	0.04	0.13	0.04	0.08	0.03	0.09	0.07	0.05	0.03	0.11
R	0.8	0.61	0.78	0.79	0.94	0.8	0.79	0.68	0.84	0.5

The mite populations from Sierra Nevada, Asturias, Rioja and Cazorla presented the highest genetic variability, while Sierra de Grazalema, Sierra Morena and Sierra de los Filabres had the mite populations with lowest variability.

**Fig. 2:**
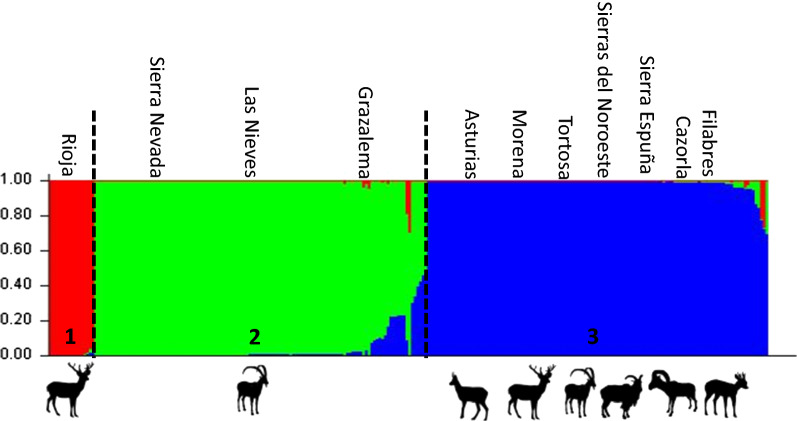
Barplot generated with software Structure 2.3.4 displaying three main clusters (*K* = 3) of *Sarcoptes*-derived genetic strains

Significant deviation from HWE was observed overall (Additional file 2: Table S2). In the Grazalema-, Los Filabres-, and Sierra Morena-derived mite populations, none of the samples supported the HWE (*P* < 0.01). The Bayesian assignment test, according to the DK method of Evanno (*K* = 3) [30], showed three main clusters of ruminant-derived mites (Fig. 2).

**Fig. 3:**
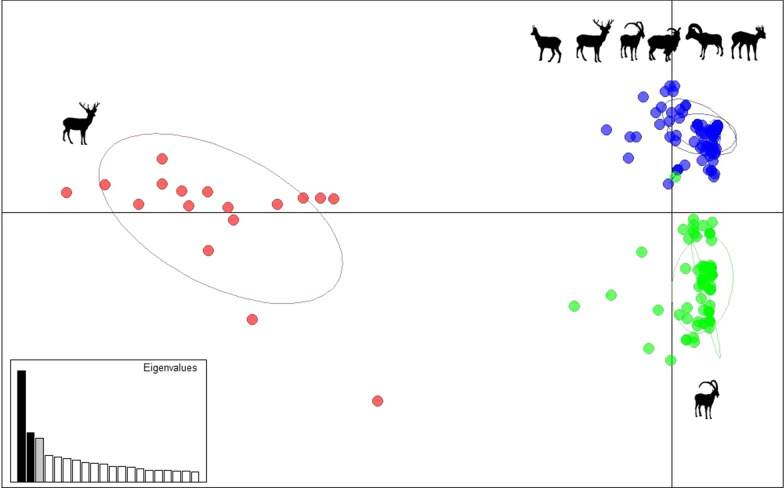
Unrooted distance-based dendrogram constructed with Populations 1.2.32 software and displayed with iTOL 5.5.1 online software representing 266 individual mites from wild herbivores. Main clusters are separated by colors (red, blue, green) with corresponding attributes (host species and origin)

**Fig. 4:**
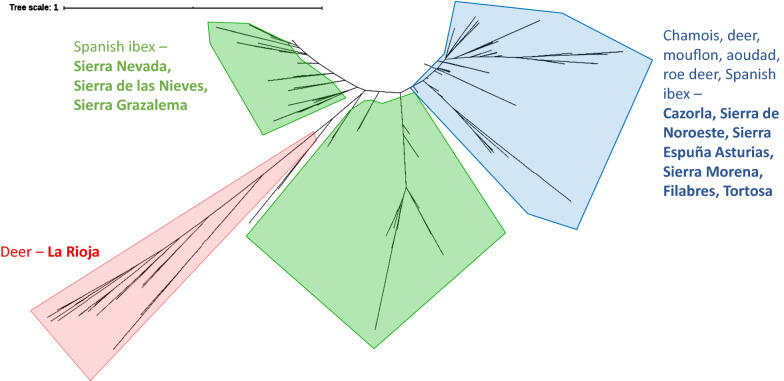
Scatter plot generated with R 4.0 software (implemented by the package adegenet 2.1.3) representing principal component analysis (PCA) of 266 mites from wild herbivores deriving from ten different geographical origins. Variance is explained by 12.4 and 5.5% of components 1 and 2, respectively. The eigenvalues of the two axes are displayed in the bar plot on the left. Label names refer to the origin of host species (see Table 1). Colors (on the red-blue-green scale) and distances display the genetic diversity

The samples within each individual cluster were consistent with an origin-based classification. The genetic distance among all the mites is displayed in the unrooted dendrogram in Fig. 3. The results of the multivariate analysis with R 4.0 are displayed in Fig. 4. The axes one and two accounted for 17.9% of the total variance. The PCA scatter plot revealed three main clusters separated by population origin: the mite population from La Rioja was the most divergent on the first axis. The other two clusters were distributed on the second axis and included the mite populations from Sierra Nevada, Sierra de las Nieves and Sierra de Grazalema, on the one hand (cluster 2) and the mite populations from Cazorla, Asturias, Sierra Morena, Sierra de los Filabres and Tortosa on the other hand (cluster 3).

**Fig. 5:**
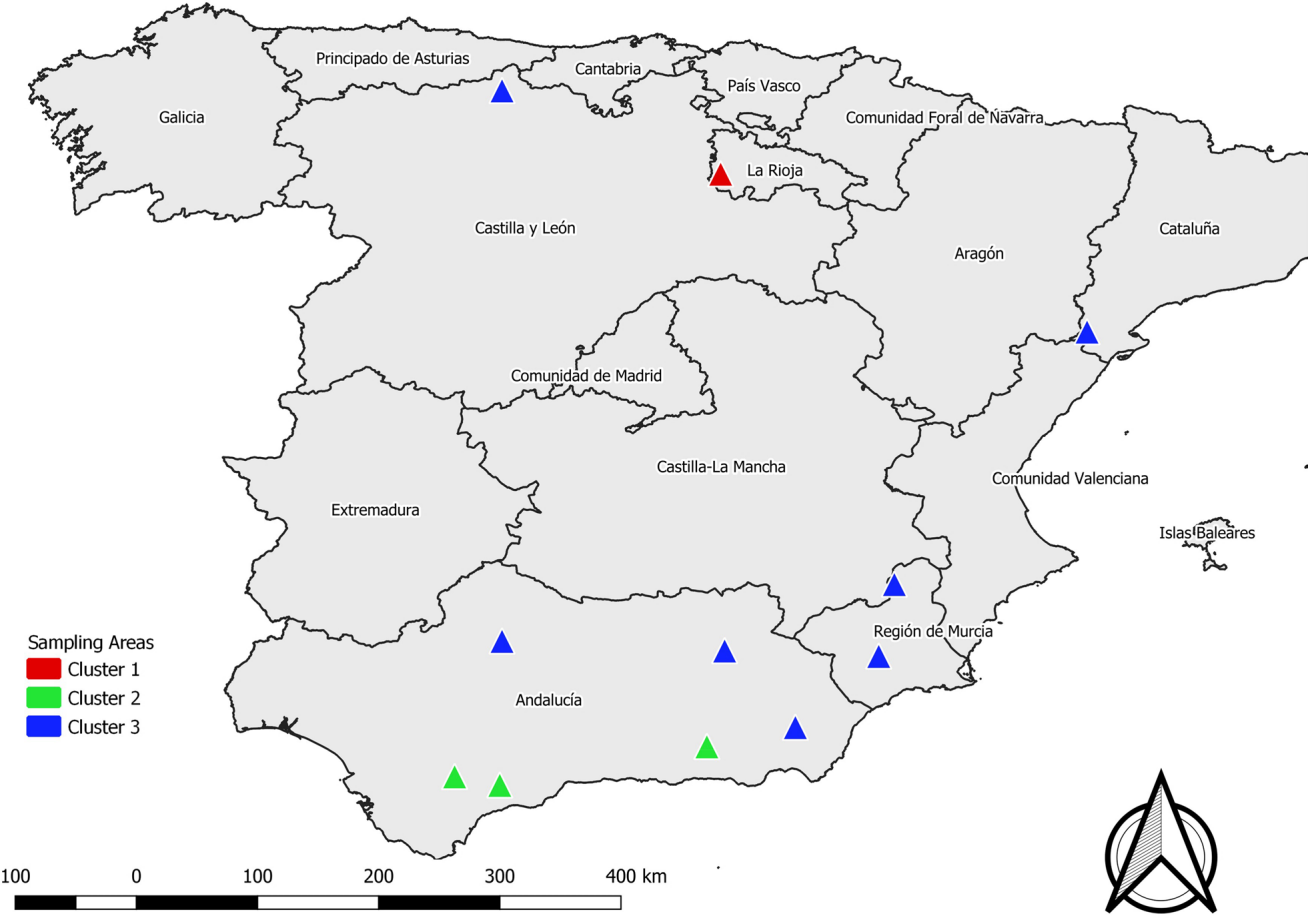
Locations of sampled animals colored accordingly to the genetic cluster analysis

Overall, the three different cluster analyses performed in agreement, regardless of HWE, in defining three groups of ruminant-derived mites, consistent with the geographical origin, displayed in Fig. 5.

## Discussion

The cluster analyses performed in our study consistently identified three main genetic clusters of *S. scabiei* in the mange-affected wild ruminant populations investigated throughout Spain. The three genetic clusters identified revealed that (i) different wild ruminant species were affected by the same *S. scabiei* strain, and (ii) circulating *S. scabiei* strains in Spain are both geographically and host-related, although (iii) geographical distance among mange-affected wild ruminant populations is not related to mite strain phylogeny, with distant populations affected by the same *S. scabiei* strain and close populations hosting different strains.

The two clusters related to a single species (La Rioja- and Sierra Nevada-derived, *C. elaphus* and *C. pyrenaica*, respectively) were also geographically limited to a single region or neighboring areas. Conversely, the third cluster (Cazorla/Asturias-derived) encompassed multi-host systems (*C. elaphus*, *C. pyrenaica*, *R. pyrenaica*, *A. lervia*, *C. capreolus* and *O. a. musimon*) and referred to different areas in the Southeast, Northwest and more recently Northeast of the Iberian Peninsula (Fig. 5).

*Sarcoptes scabiei* mites do not have free-living stages; thus main genetic mixing occurs on the same host, and skin-scale patterns of variability have been identified even in the same individual host [36, 37]. Literature data support the hypothesis that the rare exchange (and thus possible mating) of mites among different hosts may condition the genetic and epidemiological features of *S. scabiei* and its spreading patterns in different host communities [19–21]. In Spain, the spread of each putative “strain” to different sympatric host species (cluster 3) or not (clusters 1 and 2) might be dependent on (i) the host community composition and (ii) the maintenance and transmission capability of each “strain” by the individual host populations and communities. In multi-host scenarios, the more susceptible species would act as a reservoir, spreading sarcoptic mange to less susceptible hosts, which would likely not be capable of maintaining the transmission chain in the absence of the source host(s), as suspected in Cantabrian chamois (reservoir host) and red and roe deer (spillover hosts) in the Cantabrian Mountains [4, 8, 12]. A similar pattern has been reported in the Alps, where the northern chamois (*Rupicapra rupicapra*) and the Alpine ibex (*Capra ibex*) play a reservoir role, whereas the red and roe deer and European mouflon are mere spillover hosts despite their abundance and sympatry with the aforementioned native caprines [38].

The imbricated distribution of *S. scabiei* clusters (Fig. 5) in scarcely connected wild ruminant populations (in particular, see the distribution of cluster 3), in parallel with the chronology of outbreak eruption which only partially supports an “oil spot” spreading pattern of the disease amongst naïve contiguous wild ruminant populations [39], might suggest that *S. scabiei* was likely introduced by infected livestock. The high number of private alleles, particularly in the La Rioja population, might indicate low gene flow and high genetic separation among mite populations from the rest of the Iberian Peninsula. Thus, the cluster represented by deer-related *Sarcoptes* from La Rioja (Sierra de la Demanda) might imply the existence of a new *Sarcoptes*-strain that started to spread after the index case was recorded in the local ungulate population of La Rioja (see Table 1), with unknown origin. Domestic goats and sheep are well-known suitable hosts for *S. scabiei*, and cross-transmission with wild caprines has been demonstrated experimentally [25]. Transmission of *S. scabiei* at the wild–domestic interface has also been reported under natural conditions [2, 22, 25, 38]. The presence or introduction of sympatric herds of domestic goats infected with *S. scabiei* has been proposed as the origin of the first epizootic outbreak reported in the Iberian Peninsula and affecting the Spanish ibex [2], the subsequent outbreak described in Cantabrian chamois [4], and also the most recent sarcoptic mange outbreak affecting the ibex population in the Tortosa mountains [40]. However, mite isolation from goats and molecular confirmation of these suspicions were not feasible, since herds had already been treated or were not present in the area at the time of the investigation. In accordance with previous studies [19–21], the analyzed samples significantly deviated from HWE, supporting the idea that these assumptions might be inapplicable in most natural populations [31]. Moreover, He and allelic richness were low throughout all loci, implying low gene diversity. Deviations from the HWE and from random mating of mite populations might be explained by the nonrandom colonization dynamics of *S. scabiei* at the individual-host level [37] and at the subpopulation-host level (Wahlund effect).

Not all the ungulate populations or subpopulations in our sample were geographically connected, implying that gene flow between *Sarcoptes* mites was low, and in some cases, absent.

We hypothesize that, after the introduction of the mite into naïve wild ruminant populations, the parasite develops a distinctive epidemiological pattern, depending on host species composition, animal density, size and relative abundance, the sensitivity of each species, the specialization of the mite strain, and environmental and social factors, among others [10]. The role of human-driven introduction or trade of wild and domestic animals should be considered as a viable explanation for sarcoptic mange spread in different areas of the Iberian Peninsula. Given the dramatic consequences of an easier-to-manage disease in domestic livestock, such as sarcoptic mange, when introduction into naïve wild ruminant populations occurs, its importance should not be further neglected by those responsible for livestock health care and treatment [41]. This is especially relevant in those scenarios with wildlife–livestock interface, where the jump of shared pathogens may occur among susceptible and phylogenetically related host species.

## Conclusions

This study establishes the current distribution of *S. scabiei* genetic clusters (“strains”) in the main populations of free-ranging wild ruminants in Spain, pointing to a probable origin from livestock in most of the populations affected by sarcoptic mange. Scabies-free populations of wild ungulates in Spain may be exposed to infected domestic caprines in the future. Therefore, further genetic investigations, including livestock, are required to fine-tune the epidemiological role of domestic ungulates in the spread of sarcoptic mange at the wildlife–livestock interface in the Iberian Peninsula. The use of molecular tools such as the microsatellite markers applied to the genetic epidemiology of *S. scabiei* might have important health implications in wildlife restocking management plans and livestock movements, as recently proved in other wildlife species [42]. At the same time, results of this study suggest that the current and widely accepted classification of *S. scabiei* into host-related variants (*varietates*) may be insufficient to represent the complexity that growing molecular epidemiological studies in natural scenarios seem to reveal. We expect that the use of next-generation sequencing such as whole genome sequencing will soon be applied to improve the robustness and repeatability of phylogenetic studies of *S. scabiei* in animals and humans.

## Supplementary Information

**Additional file 1: Table S1.** Raw microsatellite alleles of the 10 SARMS microsatellite loci for *Sarcoptes scabiei* mites. A total of 266 mites were collected from skin samples and skin scrapings of 121 ruminants belonging to six species, namely Iberian ibex (76), Cantabrian chamois (16), red deer (18), roe deer (2), aoudad (1), and European mouflon (1), from 11 sampling locations in Spain.

**Additional file 2: Table S2. **Results of the Hardy–Weinberg equilibrium test at locus (rows) and populations (columns) showing *P* values from the Monte Carlo test. *P* values less than 0.05 (*) and 0.01 (**) are considered significant.

## Data Availability

The data sets used and analysed in the present study are included in this article. Raw data are available in the additional material.

## Abbreviations

HWE: Hardy Weinberg equilibrium
He: Expected heterozygosity
Ho: Observed heterozygosity
Sarms: *Sarcoptes* microsatellite
